# Long non-coding RNA SNHG15 in various cancers: a meta and bioinformatic analysis

**DOI:** 10.1186/s12885-020-07649-9

**Published:** 2020-11-26

**Authors:** Caizhi Chen, Yeqian Feng, Jingjing Wang, Ye Liang, Wen Zou

**Affiliations:** Department of Oncology, The Second Xiangya Hospital of Central South University, Changsha, 410000 Hunan China

**Keywords:** LncRNA SNHG15, Prognosis, Meta-analysis, Bioinformatics

## Abstract

**Background:**

The snoRNA host gene SNHG15 produces a long non-coding RNA (lncRNA) with a short half-life and has been reported to be dysregulated in multiple cancers and has recently been found to be correlated with tumour progression. Therefore, this meta-analysis was performed to evaluate the generalised prognostic role of small nucleolar RNA host gene 15 (SNHG15) in malignancies, based on variable data from different studies.

**Methods:**

Four public databases were used to identify eligible studies. The association between prognostic indicators and clinical features was extracted and pooled to estimate the hazard ratios (HRs) or odds ratios (ORs) with 95% confidence intervals (CIs). Publication bias was measured using Begg’s test and Egger’s test, and the stability of pooled results were measured using sensitivity analysis. Additionally, an online database based on The Cancer Genome Atlas (TCGA) was screened to further validate our results. Ultimately, we predicted the molecular regulation of SNHG15 based on the public databases.

**Results:**

In total, 11 studies including 1087 patients were ultimately enrolled in our meta-analysis. We found that SNHG15 overexpression was associated with worse overall survival (OS) and disease-free survival (DFS), and this was validated in the Gene Expression Profiling Interactive Analysis (GEPIA) cohort. Moreover, increased SNHG15 expression suggested advanced TNM stage and LNM, but was not associated with age, gender, or tumour size. No publication bias or instability of the results was observed. SNHG15 was significantly upregulated in seven cancers and elevated expression of SNHG15 indicated shorter OS and DFS in five malignancies based on the validation using the GEPIA cohort. Further functional prediction indicated that SNHG15 may participate in some cancer-related pathways.

**Conclusions:**

Upregulation of lncRNA SNHG15 was notably associated with worse prognosis and clinical features, suggesting that SNHG15 might serve as a novel prognostic factor in various cancers.

## Background

Cancer is a severe health problem and is the leading cause of death worldwide, with annually increasing incidence and mortality rates. According to the latest statistics reported in CA cancer journals, 1,806,590 new cancer cases and 606,520 cancer deaths are expected to occur in the United States in 2020 [1]. Although multidisciplinary treatments, such as surgery, chemotherapy, radiotherapy, targeted therapy, and immunotherapy, of malignancies have improved greatly in recent years, prognosis and early diagnosis remain extremely challenging [2]. As such, there is an urgent need to identify innovative and effective targets for investigating the signalling pathways in tumours, which may ultimately play an indispensable role in therapeutic decision-making for cancer patients.

Long non-coding RNAs (lncRNAs), which were initially speculated to be transcriptional noise with no specific biological function, have emerged as a novel category of non-coding RNAs (ncRNAs) exceeding 200 nucleotides in length that are transcribed by RNA polymerase II but do not encode proteins due to the lack of an open reading frame [3]. Nonetheless, a growing body of work has demonstrated that aberrant expression of lncRNAs is correlated with biological processes, including tumour progression, angiogenesis, metastasis, and invasion, indicating that lncRNAs can serve as tumour suppressors or oncogenes for cancer control [4, 5]. Recently, small nucleolar RNA host gene 6 (SNHG6), linc00152, and opa-interacting protein 5 antisense RNA 1(OIP5-AS1) have been identified as potential prognostic biomarkers involved in the modulation of tumour-related genes and specific molecular mechanisms in human cancers [6–8].

Small nucleolar RNA host gene 15 (SNHG15), which is located at 7p13 and is 860 base pairs long, was initially reported as a lncRNA with a short half-life [9]. As a tumour oncogene, lncRNA SNHG15 functions as a competing endogenous RNA (ceRNA) to sponge miR-153, miR-38, miR-141, and miR-141-3p, which consequently promotes cell proliferation, migration, invasion, autophagy, and cisplatin resistance in glioma, breast cancer, osteosarcoma, and hepatocellular carcinoma [10–13]. Furthermore, SNHG15 enhances tumour development or drug resistance in glioblastoma multiforme, colorectal carcinoma, and prostate cancer through the SNHG15/CDK6/miR-627, SNHG15/miR-141/SIRT1/Wnt/β-catenin, SNHG15/miR-338-3p/FKBP1A, and SNHG15/miR-338-3p/FOS-RAB14 axes [14–17]. Additionally, it was found that SNHG15 could facilitate cell proliferation, invasion, and drug resistance in colorectal cancer by acting as a bifunctional MYC-regulated noncoding locus encoding an lncRNA that interacts with AIF. Similarly, it was demonstrated that SNHG15 promoted tumour progression in colon cancer by stabilising the transcription factor Slug [18, 19]. However, only one report has found SNHG15 to be downregulated in thyroid cancer tissue samples and cells, suggesting its role as a tumor suppressor and the reduced expression of SNHG15 enhanced cell proliferation, migration, and invasion in vitro [20].

Collectively, most studies have demonstrated that SNHG15 is involved in gene regulation by acting as an oncogene in various malignancies, and its elevated expression might be associated with the prognosis and clinicopathological parameters of gastric cancer, hepatocellular carcinoma, lung cancer, non-small cell lung cancer, renal cell carcinoma, pancreatic ductal adenocarcinoma, breast cancer, papillary thyroid carcinoma, colorectal cancer, and epithelial ovarian cancer [21–31].

However, given the discrepancies between published studies, the small number of patient samples, and the different detection methods used, the prognostic value of SNHG15 remains unclear at this time. Therefore, we conducted this meta-analysis and bioinformatic validation to determine whether SNHG15 could be used as a non-invasive prognostic marker of tumours and attempted to reach a consensus regarding the prognostic value of this gene.

## Methods

### Search strategies for eligible literature

Relevant articles that investigated the association between SNHG15 expression and the clinical outcomes of cancer patients were searched using PubMed, Web of Science, Embase, and the Cochrane Library through February 26, 2020. Three domains of keywords in multiple combinations were utilised as search subjects as follows: (“long noncoding RNA” OR “lncRNA”) AND (“SNHG15” OR “small nucleolar RNA host gene 15”) AND (“Cancer” OR “Cancers” OR “Tumors” OR “Tumor” OR “Malignancy” OR “Malignancies” OR “Neoplasia” OR “Neoplasias” OR “Neoplasm” OR “Malignant Neoplasms” OR “Malignant Neoplasm” OR “Neoplasm, Malignant” OR “Neoplasms, Benign” OR “Benign Neoplasm” OR “Neoplasms, Malignant” OR “Benign Neoplasms” OR “Neoplasm, Benign”). Further, a manual search was conducted to avoid overlooking eligible papers by screening the title and abstracts of papers from the references lists of pertinent articles.

### Inclusion and exclusion criteria

All enrolled studies were assessed by two independent investigators, and disagreements were resolved by reaching a consensus after discussion with the third author. Articles that met the following criteria were enrolled in our study: (1) original articles investigating the role of SNHG15 in cancers that were definitively diagnosed by histopathology; (2) samples were cancer tissue and adjacent normal tissue; (3) detection method was qRT-PCR; (4) clinical features, including age, gender, tumour size, TNM stage, lymph node metastasis or distant metastasis, and prognostic indicators, such as overall survival (OS), disease-free survival (DFS), or progression-free survival (PFS), were reported in the paper; (5) patients were categorised into increased and decreased SNHG15 expression groups based on a cut-off value, and the number of patients in these two groups was explicitly stated; (6) hazard ratios (HRs) and 95% confidence intervals (CIs) were reported by multivariate analysis from the articles or were available to be indirectly calculated via Kaplan-Meier (K-M) curves; and (7) the language of the article was English.

Exclusion criteria: (1) studies exploring other lncRNAs or those that were not related to cancers; (2) duplicate articles; (3) other literature types, such as reviews, letters, conference abstracts, meta-analyses, case reports, retractions, etc.; (4) articles focussed on biological functions; and (5) lack of sufficient data for HR and 95% CI extraction.

### Data extraction and quality evaluation

The main information from eligible studies was extracted as follows: first author, publication year, country, cancer type, sample type, sample size (high/low), cut-off value for SNHG15 expression, assay method, survival (OS/RFS/PFS), HR availability, HR (95% CI) with its *P* value, follow-up months, and Newcastle-Ottawa Scale (NOS) scores. If survival rates were not obtained from multivariate analysis, the survival HR (95% CI) was indirectly retrieved from K-M curves by using Engauge Digitiser software. The quality of the enrolled studies was assessed using the NOS score with a range from 0 to 9, and a score greater than 6 was considered as qualified literature.

### Validation of bioinformatics database

Gene Expression Profiling Interactive Analysis (GEPIA), which is based on The Cancer Genome Atlas (TCGA), was performed to further verify the abnormal expression of SNHG15 among cancer tissues and to match TCGA normal and GTEx data among various neoplasms with *P* < 0.01 as the cut-off value. Survival plots of the correlation between SNHG15 expression and OS and DFS were retrieved as K–M curves based on different cancer datasets online.

### Functional prediction of SNHG15

We identified SNHG15 relevant ceRNA regulations by starBase, LncBase Predicted v.2, miRDB, TargetScan, miRTarBase, mirDIP and used Cytoscape to construct visualized ceRNA network.

### Statistical analysis

Stata (Version 12.0) was used to analyse all the data extracted from the articles included in this study, and a *P* value < 0.05 indicated a significant difference. The HR and odds ratio (OR), with their corresponding 95% CIs, were utilised to analyse the association between SNHG15 expression and prognostic indicators (OS/DFS) and clinical features, respectively. When HR/OR > 1 and a 95% CI not including 1 were observed in the results, this implied that patients with SNHG15 overexpression had a worse prognosis and advanced clinicopathological parameters. Cochran’s Q and I^2^ statistics were determined to measure the heterogeneity across all enrolled studies. A random-effect model was applied with the existence of marked heterogeneity as I^2^ > 50% and *P* < 0.10, otherwise a fixed-effect model was used. Begg’s and Egger’s tests were quantitatively conducted to detect underlying publication bias. Accordingly, sensitivity analysis was used to evaluate the stability of the results.

## Results

### Screening process of published literature

**Fig. 1:**
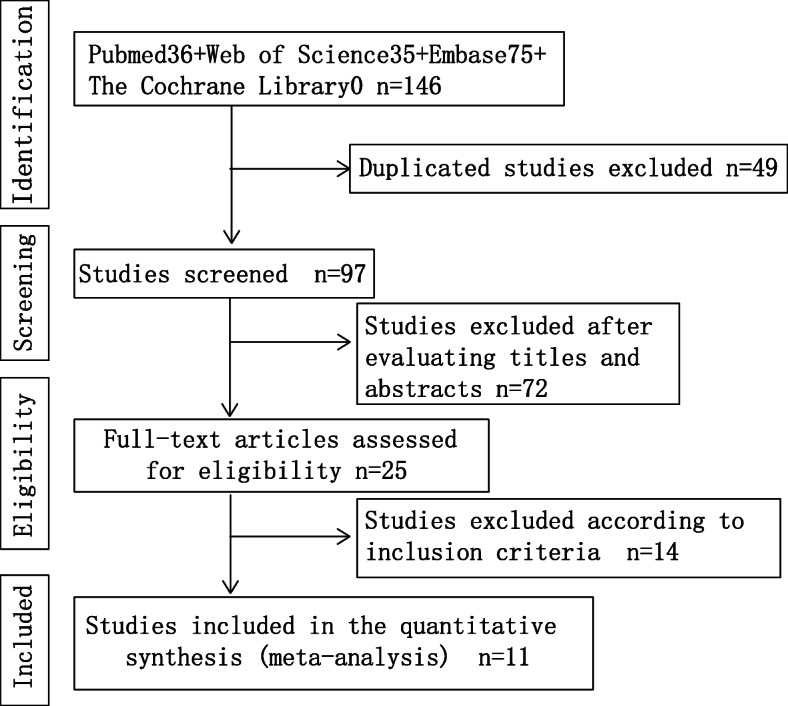
Flow diagram of the meta-analysis

A systematic database search of the literature was conducted, including initially pertinent publications regarding the correlation between SNHG15 and cancers, in PubMed (*n* = 36), Web of Science (*n* = 35), Embase (*n* = 75), and the Cochrane Library (*n* = 0). After initially removing duplicates (*n* = 49), the titles and abstracts of the remaining studies (*n* = 97) were assessed. Seventy-two studies were removed due to being irrelevant topics, reviews, case reports, and conference abstracts. Next, 25 full-text articles were assessed for eligibility. Among them, nine were removed due to a focus on the functional exploration of SNHG15, two were excluded due to a lack of prognostic data, and three articles were excluded due to unclear group numbers. Ultimately, 11 articles containing sufficient data of both survival and clinical features were enrolled in our meta-analysis. Figure 1 presents the detailed selection process for qualified publications.

### Characteristics of the enrolled studies

Eleven studies performed in China, including a total of 1087 patients, that were published from 2016 to 2019 were included. Regarding cancer types, three studies explored lung carcinoma, including one lung cancer and two NSCLC, while the others investigated gastric cancer, hepatocellular carcinoma, renal cell carcinoma, pancreatic ductal adenocarcinoma, breast cancer, papillary thyroid cancer, colorectal cancer, and epithelial ovarian cancer. All samples were cancer tissues and adjacent normal tissues, and the detection assay was qRT-PCR in all cases. Patients were classified into high and low SNHG15 expression groups, and most studies used the median expression level as the cut-off value, except for one study which utilised the mean value and one which did not provide a cut-off value. All studies reported OS, while only two referred to DFS and one mentioned PFS. Regarding HR with 95% CI availability, there were five instances where this could be obtained directly from the papers, and for the remaining cohorts, this was retrieved from K-M curves using Engauge Digitiser software. The follow-up time ranged from 40 to 180 months. The quality of the enrolled studies was assessed by NOS, with scores ranging from 6 to 8. The main features of the enrolled studies are listed in Table 1.

**Table 1:** Characteristics of the included studies

First author	Year	Country	Cancer type	Sample	Sample size (high/low)	Cut-off value	Method	Survival	HR availability	HR(95%CI) *P* value	Follow-up months	NOS
Chen	2016	China	gastric cancer	tissue	106 (53/53)	Median	qRT-PCR	OS/DFS	reported	2.928 (1.304–6.575) 0.009 /	40	8
										2.399 (1.377–4.177) 0.002		
Zhang	2016	China	HCC	tissue	152 (77/75)	Median	qRT-PCR	OS	reported	2.247 (1.331–6.255) 0.001	70	7
Cui	2018	China	lung cancer	tissue	55 (27/28)	NM	qRT-PCR	OS	reported	2.234 (1.033–4.829) 0.041	80	7
Dong	2018	China	NSCLC	tissue	49 (23/26)	Mean	qRT-PCR	OS/DFS	K-M curve	1.878 (0.840–4.200) 0.125/	120	7
										2.153 (1.010–4.590) 0.047	80	
Du	2018	China	RCC	tissue	96 (48/48)	Median	qRT-PCR	OS	K-M curve	1.022 (0.480–2.180) 0.953	160	6
Guo	2018	China	PDAC	tissue	171 (82/89)	Median	qRT-PCR	OS	reported	3.251 (1.177–6.362) 0.004	60	7
Jin	2018	China	NSCLC	tissue	35 (20/15)	Median	qRT-PCR	OS	K-M curve	1.414 (0.380–5.260) 0.606	60	6
Kong	2018	China	breast cancer	tissue	58 (29/29)	Median	qRT-PCR	OS	K-M curve	2.126 (0.900–5.020) 0.086	60	6
Wu	2018	China	PTC	tissue	92 (50/42)	Median	qRT-PCR	OS	K-M curve	1.081 (0.350–3.340) 0.892	60	6
Huang	2019	China	colorectal cancer	tissue	91 (46/45)	Median	qRT-PCR	OS	reported	2.731 (1.005–7.424) 0.049	84	7
Qu	2019	China	EOC	tissue	182 (73/109)	Mean	qRT-PCR	OS/PFS	K-M curve	1.918 (1.210–3.040) 0.006/	60	8
										1.844 (1.180–2.880) 0.007		

*HCC* hepatocellular carcinoma; *NSCLC* non-small cell lung cancer; *RCC* renal cell carcinoma; *PDAC* pancreatic ductal adenocarcinoma; *PTC* papillary thyroid cancer; *EOC* epithelial ovarian cancer; *NM* not mention; *OS* overall survival; *DFS* disease-free survival; *PFS* progression-free survival; *K-M curve* Kaplan–Meier curve; *qRT-PCR* quantitative real time polymerase chain reaction; *NOS* Newcastle-Ottawa Scale

### Association between SNHG15 expression and clinical outcomes

**Fig. 2:**
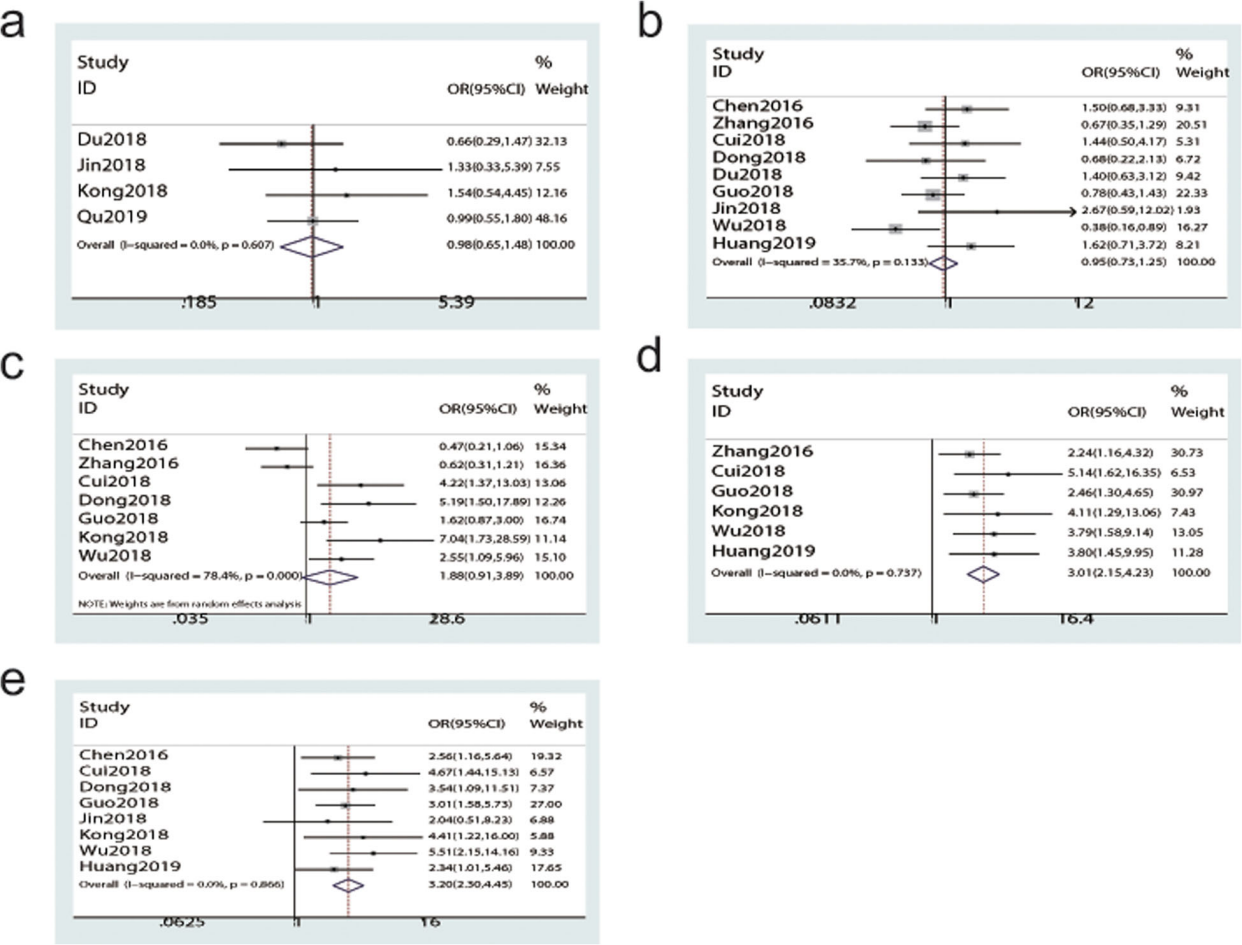
Forest plots evaluating the association between SNHG15 expression and clinical features. **a** Age, **b** Gender, **c** Tumour size, **d** TNM stage, **e** Lymph node metastasis

The correlation between SNHG15 expression and clinical features was investigated by calculating the pooled OR and 95% CI of age, gender, tumour size, TNM stage and LNM. Results indicated that SNHG15 overexpression was not significantly associated with age (< 60 vs. ≥60, OR = 0.98, 95% CI: 0.65–1.48, *P* = 0.912, Fig. 2a), gender (male vs. female, OR = 0.95, 95% CI: 0.73–1.25, *P* = 0.728, Fig. 2b), tumour size (large vs. small, OR = 1.88, 95% CI: 0.91–3.89, *P* = 0.087, Fig. 2c). However, a significant association was observed between increased SNHG15 expression and advanced clinical features, including TNM stage (III-IV vs. I-II, OR = 3.01, 95% CI: 2.15–4.23, *P* < 0.001, Fig. 2d) and LNM (positive vs. negative, OR = 3.20, 95% CI: 2.30–4.45, P < 0.001, Fig. 2e). Four fixed-effect models and one random-effect model were adopted for the data with low heterogeneity (0–35.7%) and significant heterogeneity (78.4%), respectively, and the details are shown in Table 2.

**Table 2:** Results of the association between SNHG15 and clinicopathological outcomes

						Heterogeneity		
outcome	studies(n)	OR	95%CI	*P* value	Model	Chi2	I2	*P* Value
age(< 60 vs ≥60)	4	0.98	0.65–1.48	0.912	Fixed	1.84	0%	0.607
gender (male vs female)	9	0.95	0.73–1.25	0.728	Fixed	12.44	35.7%	0.133
TNM stage (III-IV vs I-II)	6	3.01	2.15–4.23	0.000	Fixed	2.76	0%	0.737
lymph node metastasis (positive vs negative)	8	3.20	2.30–4.45	0.000	Fixed	3.20	0%	0.866
tumor size (large vs small)	7	1.88	0.91–3.89	0.087	Random	27.76	78.4%	0.000
overall survival	11	1.95	1.53–2.49	0.000	Fixed	6.43	0%	0.778
disease-free survival	2	2.31	1.48–3.61	0.000	Fixed	0.05	0%	0.822

**Fig. 3:**
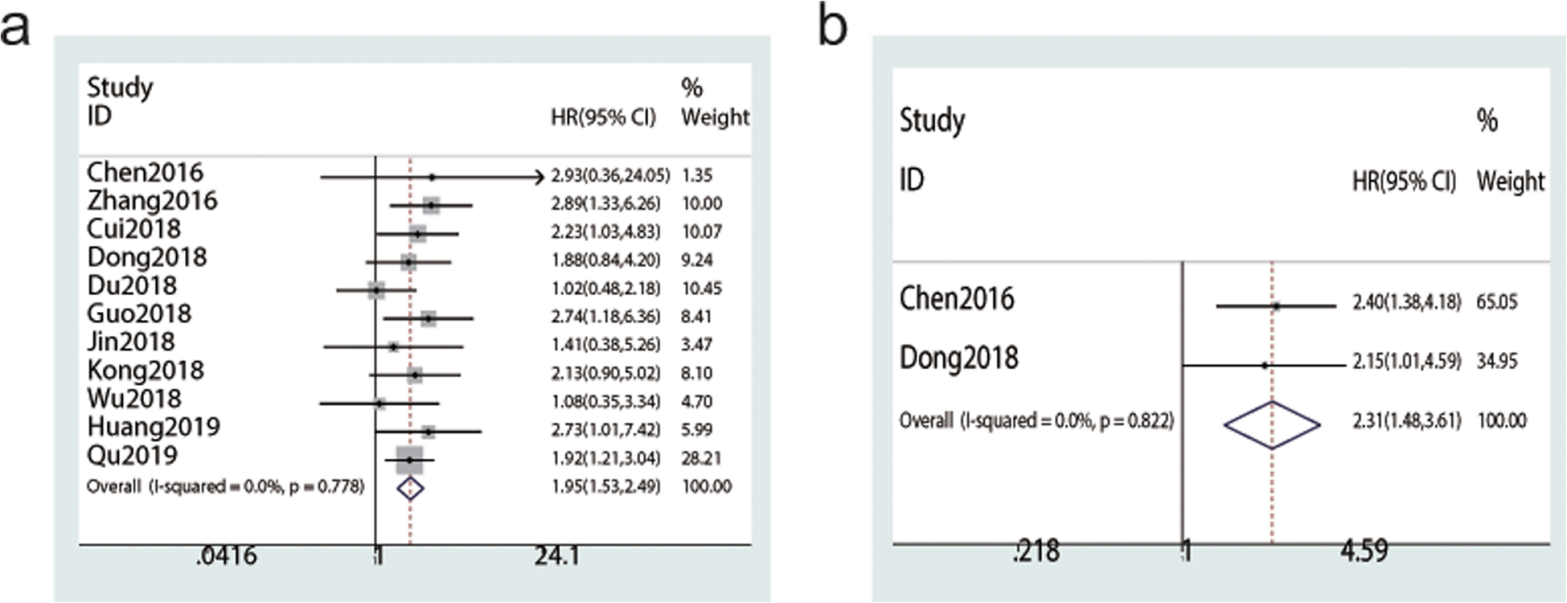
Forest plots assessing the association between SNHG15 expression and prognosis. (**a**) OS. (**b**) DFS

To further demonstrate whether SNHG15 could serve as a prognostic predictor in various cancers, we explored the association between elevated SNHG15 expression and survival indicators (OS/DFS). All enrolled studies reported the OS and a forest plot revealed that the pooled HR and 95% CI were 1.95 (1.53–2.49) by using the fixed-effect model (I^2^ = 0%, *P* = 0.778), suggesting that SNHG15 overexpression indicated worse OS (*P* < 0.001, Fig. 3a). Similarly, as shown in Fig. 3b, no significant heterogeneity in DFS was observed in two studies (I^2^ = 0%, *P* = 0.822); therefore, the fixed-effect model was employed. The pooled results revealed that increased SNHG15 expression was significantly associated with worse DFS (HR = 2.31, 95% CI: 1.48–3.61, *P* < 0.001, Fig. 3b). Given that no obvious heterogeneity was observed in the results, we did not perform subgroup analysis. Additionally, we only analysed publication bias for OS given that only two studies reported DFS. More detailed information is provided in Table 2.

### Publication bias and sensitivity analysis for prognosis

**Fig. 4:**
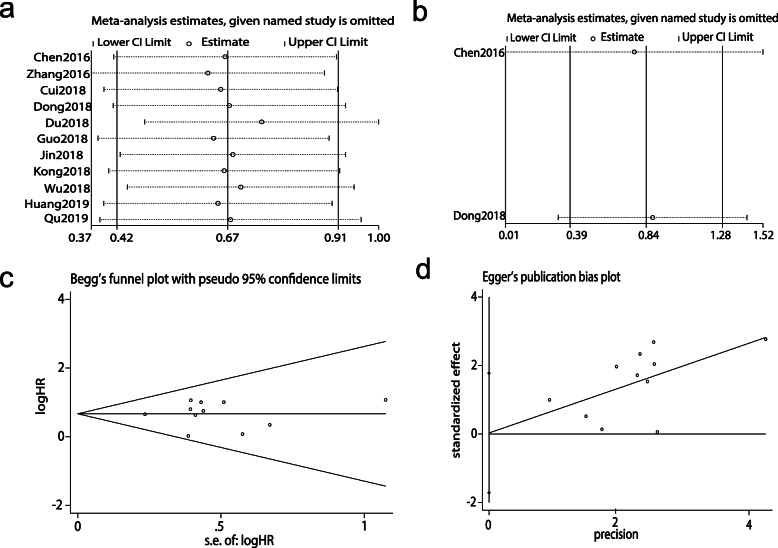
Forest plots of sensitivity analysis and publication bias. **a** Sensitivity analysis of the relationship between SNHG15 expression and OS. **b** Sensitivity analysis of the relationship between SNHG15 expression and DFS, the solid line represents the meta-analysis fixed-effect estimates, in which the 95% CI is represented by the width of the dotted horizontal line. The circle represents each study. **c** Begg’s forest plot of publication bias for OS. **d** Egger’s forest plot of publication bias for OS, HR, s.e.; standard error. Each point represents a separate study

The pooled HR for OS and DFS was not influenced after removing any single study, one by one, in the sensitivity analysis, indicating the reliability and stability of our results (Fig. 4a-b). Furthermore, Begg’s test and Egger’s test (*P* = 0.938 and *P* = 0.970, respectively) both quantitatively revealed that there was no significant publication bias in OS (Fig. 4c-d).

### Validation of the results in the GEPIA database

**Fig. 5:**
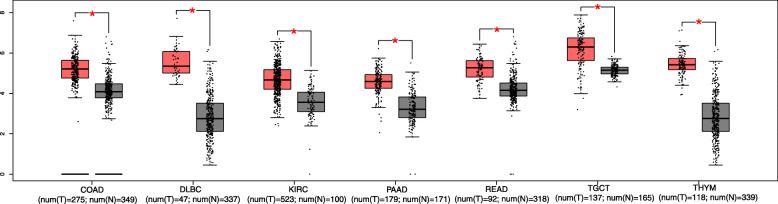
SNHG15 expression in seven types of cancer vs. normal tissue. “*“丨Log2Fold Chance丨 > 1 and *P* < 0.01. The red box plots represent SNHG15 expression in cancer tissues and the grey box plots represent SNHG15 expression in normal tissues

**Fig. 6:**
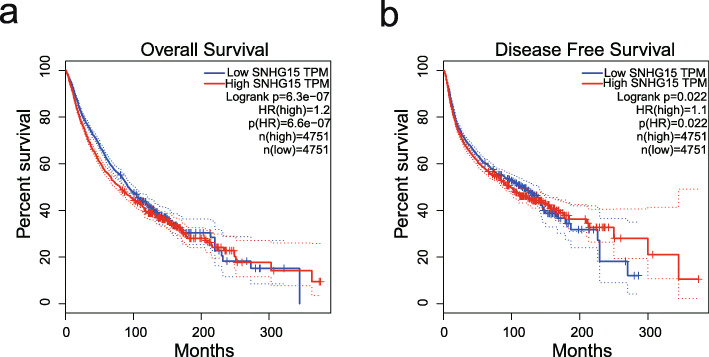
The relationship between SNHG15 expression and cancer patient prognosis in the GEPIA cohort. **a** OS plots based on SNHG15 expression in 33 types of cancer (n (low) =4751 vs n (high) = 4751). **b** DFS plots based on SNHG15 expression in 33 types of cancer (n (low) = 4751 vs n (high) = 4751)

**Fig. 7:**
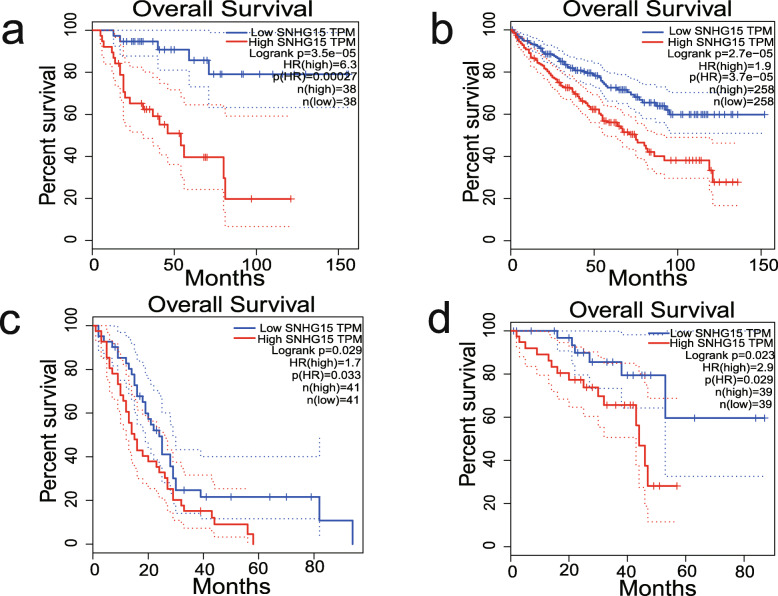
Validation of the prognostic value of SNHG15 based on the TCGA database. **a** OS plots of SNHG15 in ACC. **b** OS plots of SNHG15 in KIRC. **c** OS plots of SNHG15 in MESO. **d** OS plots of SNHG15 in UVM

**Fig. 8:**
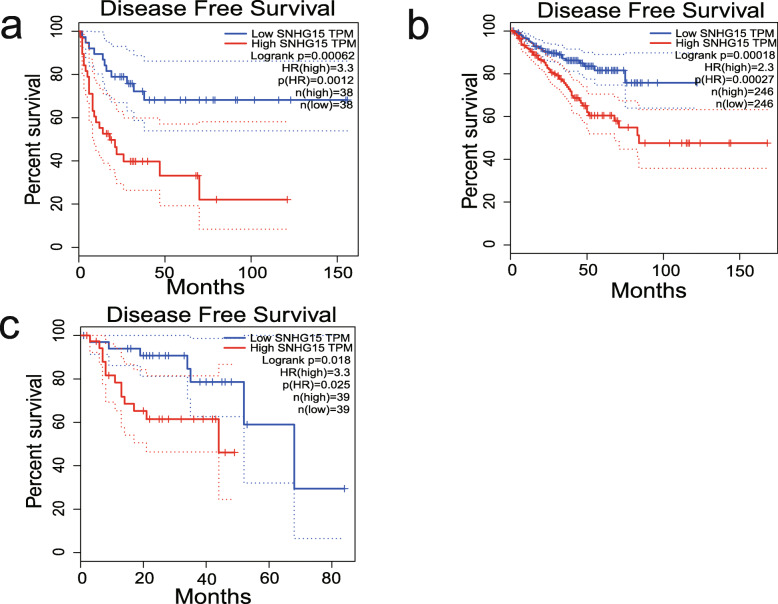
Validation of the prognostic value of SNHG15 based on the TCGA database. **a** DFS plots of SNHG15 in ACC. **b** DFS plots of SNHG15 in PRAD. **c** DFS plots of SNHG15 in UVM

The GEPIA database was used to further validate our results. In terms of SNHG15 dysregulation, SNHG15 overexpression was identified in colon adenocarcinoma (COAD), lymphoid neoplasm diffuse large B-cell lymphoma (DLBC), kidney renal clear cell carcinoma (KIRC), pancreatic adenocarcinoma (PAAD), rectum adenocarcinoma (READ), testicular germ cell tumours (TGCT), and thymoma (THYM) (Fig. 5). Regarding the association between SNHG15 expression and prognosis, survival plots assessing 9502 patients with 33 types of malignancies in the GEPIA cohort divided into high and low expression groups based on median value revealed that SNHG15 upregulation was associated with worse OS and DFS (Fig. 6), confirming the results of our meta-analysis. Furthermore, increased SNHG15 expression was correlated with worse OS in adrenocortical carcinoma (ACC), KIRC, mesothelioma (MESO), uveal melanoma (UVM), and worse DFS in ACC, prostate adenocarcinoma (PRAD), UVM (log-rank *P* < 0.05) (Figs. 7 and 8). These results support our conclusions and indicate that SNHG15 could be a novel prognostic biomarker for various cancers.

### Prediction of SNHG15 function

**Fig. 9:**
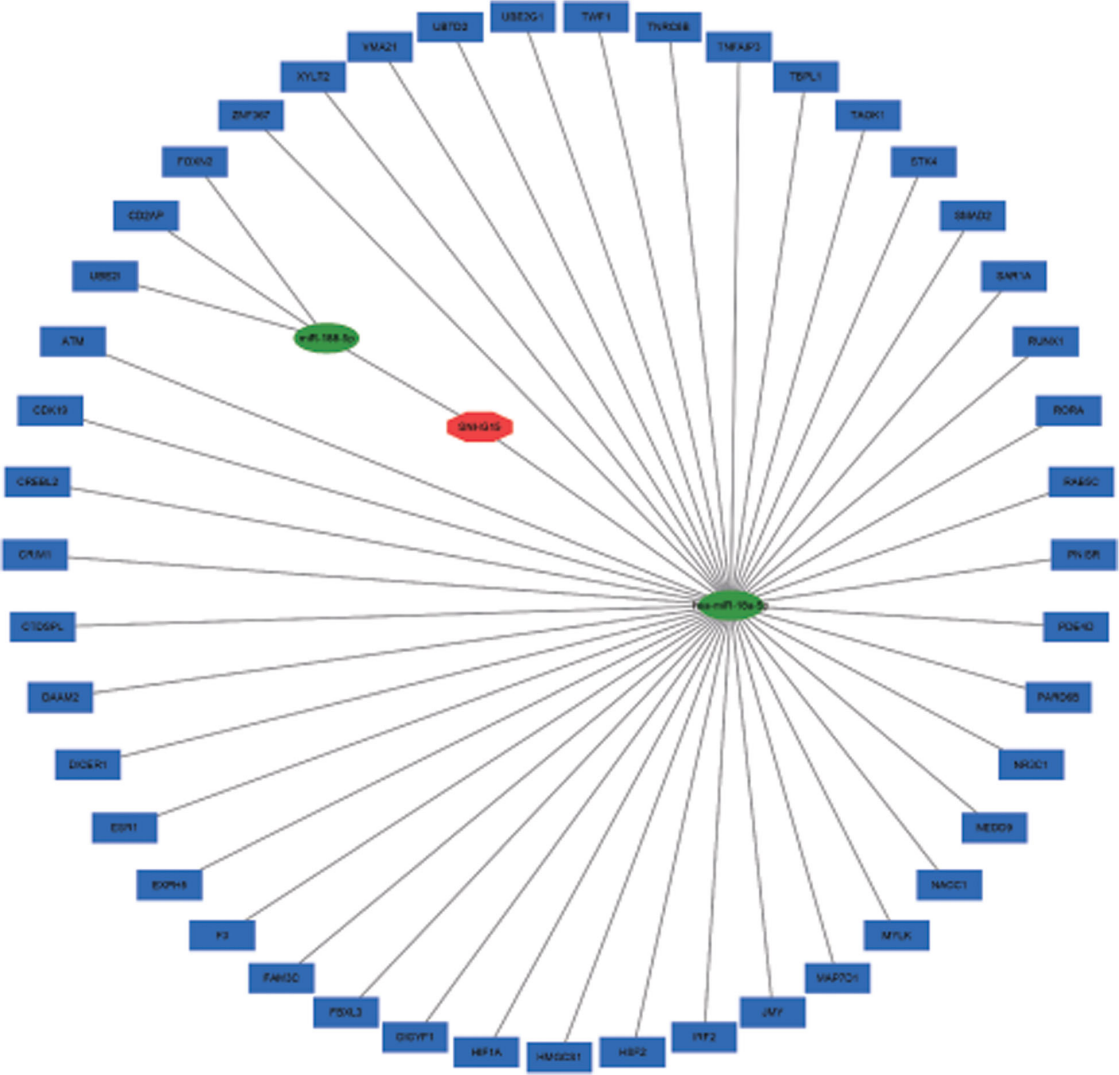
Construction of SNHG15-mediated ceRNA network. SNHG15-mediated ceRNA network is comprised of 2 miRNAs and 45 mRNAs. Blue rectangle represents mRNA, green oval stands for miRNA, red polygon is SNHG15

To further understand the molecular mechanism of SNHG15 overexpression affecting the prognosis of various cancers, we predicted its possible biological function and involved signaling pathways of SNHG15 using six online databases. First, the ceRNA regulations for SNHG15 were identified through starBase, LncBase Predicted v.2, miRDB, TargetScan, miRTarBase, mirDIP online prediction, and then a SNHG15-miRNA-mRNA network was constructed by utilizing cytoscape software (Fig. 9).

## Discussion

LncRNAs were initially thought to be “transcriptional noise” or “junk DNA” and received little attention in the previous few decades [32]. However, as next-generation genome-wide sequencing and microarrays have been widely applied in clinical settings in recent years, new research has suggested that aberrant expression of lncRNAs may promote or suppress tumour growth, leading to carcinogenesis and cancer progression [33, 34]. For example, some lncRNAs, such as NOC2L-4.1, TUG1, and MALAT1, are well established to promote tumour growth, while other lncRNAs, such as ASMTL-AS1, LINC02381, and LINC02499 have been found to inhibit tumour progression [35–40].

SNHG15, a promising new cancer-related lncRNA, has been found to be upregulated in a diverse array of malignant tumours. It has been demonstrated that elevated expression of SNHG15 is significantly related to tumour size, TNM stage, and lymph node metastasis in pancreatic cancer patients [41]. However, the definitive prognostic role of this gene was previously unclear. In our meta-analysis, we investigated the potential association between SNHG15 expression and prognostic attributes and clinicopathological parameters by integrating data from 11 studies. We found that SNHG15 overexpression increased the risk of shorter OS and DFS with no conspicuous heterogeneity. Simultaneously, we demonstrated that patients with increased SNHG15 expression were more likely to develop advanced TNM stage and positive lymph node metastasis, while these effects were not associated with age, sex, or tumour size. Additionally, no evident publication bias in OS was identified throughout the study, and the robustness of the results was verified via sensitivity analysis. Furthermore, validating the TCGA datasets revealed that high SNHG15 expression levels were observed in COAD, DLBC, KIRC, PAAD, READ, TGCT, and THYM. We also evaluated the TCGA cohort to confirm the prognostic role of SNHG15 in various cancers, and the elevated expression of SNHG15 in 33 types of tumour tissues was associated with worse OS and DFS. In some cancers, including ACC, KIRC, MESO, and UVM, SNHG15 overexpression indicated shorter OS. Moreover, survival plots revealed that ACC, PRAD, and UVM patients with SNHG15 upregulation exhibited worse DFS. Taken together, these results indicate that SNHG15 could serve as a biological modulator and novel biomarker of poor prognosis in cancer patients.

LncRNAs can indirectly regulate the expression and function of target genes via ceRNA. Thus, we constructed a ceRNA network to assess the potential function and molecular mechanism of SNHG15 in cancers. The results of our functional analysis demonstrated that target genes indirectly affected by SNHG15 may be involved in some of these signaling pathways, and promoted the proliferation, invasion and metastasis of tumour cells. Accordingly, the potential molecular mechanism involved in tumour progression may be investigated in the future to better understand the association between altered SNHG15 expression and poor prognosis (Table 3). In gastric cancer, SNHG15 upregulation promotes cell proliferation and invasion by modulating the expression of MMP2/MMP9 [21]. In lung cancer, it has been reported that SNHG15 overexpression enhances tumour occurrence and development by targeting miRNA-211-3p to regulate cell proliferation and migration in vitro [23]. Similarly, in non-small cell lung cancer, two studies have demonstrated that SNHG15 knockdown suppresses tumorigenesis by inhibiting the expression of EMT, MMP2, and MMP9 and regulating the miR-486/CDK14 axis [24, 25]. Meanwhile, another study has identified that SNHG15 facilitates renal cell carcinoma invasion and migration through the NF-κB signalling pathway and by inducing the EMT process [26]. In breast cancer, it has been shown that SNHG15 functions as a ceRNA to sponge miR-211-3p, thereby promoting cell proliferation, migration, and invasion and inhibiting apoptosis [28]. Additionally, SNHG15 has been reported to act as a ceRNA to modulate the miR-200a-3p/YAP1-Hippo axis in papillary thyroid carcinoma [29]. Consistently, functional assays have revealed that upregulation of SNHG15 facilitates the migration, invasion, proliferation, and chemoresistance of epithelial ovarian cancer cells [31]. However, the signalling pathways involved in in HCC, PDAC, and colorectal cancer remain unclear; therefore, additional studies are needed to explore the potential mechanisms by which SNHG15 expression predicts survival across diverse malignancies [22, 27, 30].

**Table 3:** Summary of SNHG15 with their aberrant expression,biological functions, and related signaling pathways

Study	Cancer	Expression	biological functions	related signaling pathways
Chen 2016 [21]	gastric cancer	upregulation	promote cell proliferation and invasion, inhibit apoptosis	MMP2/MMP9
Cui 2018 [23]	lung cancer	upregulation	promote cell proliferation,invasion	microRNA-211-3p
Dong 2018 [24]	non-small cell lung cancer	upregulation	promote cell proliferation, invasion and metastasis, inhibit apoptosis.	EMT/MMP2/MMP9
Du 2018 [26]	renal cell carcinoma	upregulation	promote cell proliferation, invasion and migration	EMT/NF-κB
Jin 2018 [25]	non-small cell lung cancer	upregulation	promote cell proliferation, induce apoptosis and cycle arrest at G0/G1 phase	miR-486/CDK14
Kong 2018 [28]	breast cancer	upregulation	promote cell proliferation, migration,invasion and induce apoptosis	miR-211-3p/EMT
Wu 2018 [29]	papillary thyroid cancer	upregulation	promote cell growth and migration	miR-200a-3p/YAP1-Hippo
Qu 2019 [31]	epithelial ovarian cancer	upregulation	promote cell migration, invasion, proliferation and induce chemoresistance	–

Several key points from our paper should be noted. First, our meta-analysis was the first study to exhaustively investigate the association between SNHG15 expression and clinical outcomes in cancer patients. In addition, only one random-effect model was employed in the analysis, indicating that the results are credible and accurate. Furthermore, we determined rigorous inclusion and exclusion criteria to enrol only high-quality studies.

Nonetheless, several limitations in our study should be considered. First, all the included subjects were from China, with small case numbers of certain cancer types and a small sample size, which led to our results being only applicable to Asia. To address this, we further validated these results using the GEPIA database to support our conclusion as broadly as possible. Further, HRs with 95% CIs were retrieved from K-M curves in six studies, which may inevitably exaggerate the prognostic value of SNGH15 and introduce bias. Moreover, the lack of articles with negative results may have caused an overestimation of the clinical value of this gene. Additionally, the inconsistent cut-off values may introduce heterogeneity among the studies.

## Conclusions

Taken together, despite the above limitations, our study revealed that SNHG15 overexpression is significantly associated with unfavourable prognosis and advanced clinical features. However, high-quality studies with standardised methods and larger sample sizes from different countries are still needed to confirm our results.

## Data Availability

All data used in this study are included in this published article.

## Abbreviations

ACC: adrenocortical carcinoma
ceRNA: competing endogenous RNA
CI: confidence interval
COAD: colon adenocarcinoma
DFS: disease-free survival
DLBC: lymphoid neoplasm diffuse large B-cell lymphoma
EMT: Epithelial-Mesenchymal Transition
GEPIA: Gene Expression Profiling Interactive Analysis
GTEx: Genotype-Tissue Expression
HCC: hepatocellular carcinoma; HR: hazard ratio
K-M: Kaplan-Meier
KIRC: kidney renal clear cell carcinoma
lncRNA: long non-coding RNA
LNM: lymph node metastasis
MESO: mesothelioma
ncRNA: non-coding RNA
NOS: Newcastle-Ottawa Scale
NSCLC: non-small cell lung cancer
OIP5-AS1: opa-interacting protein 5 antisense RNA 1
OR: odds ratio
OS: overall survival
PAAD: pancreatic adenocarcinoma
PDAC: pancreatic ductal adenocarcinoma
PFS: progression-free survival
PRAD: prostate adenocarcinoma
qRT-PCR: Real-time quantitative-Polymerase Chain Reaction
READ: rectum adenocarcinoma
RFS: relapse free survival
s.e.: standard error
SNHG15: small nucleolar RNA host gene 15
SNHG6: small nucleolar RNA host gene 6
snoRNA: small nucleolar RNA
TCGA: The Cancer Genome Atlas
TGCT: testicular germ cell tumours
THYM: thymoma
TNM: Tumor Node Metastasis
UVM: uveal melanoma

## References

[1] Siegel RL, Miller KD, Jemal A (2020). Cancer statistics, 2020. CA Cancer J Clin.

[2] Siegel RL, Miller KD, Jemal A (2017). Cancer statistics, 2017. CA Cancer J Clin.

[3] Zhang Y, Tao Y, Liao Q (2018). Long noncoding RNA: a crosslink in biological regulatory network. Brief Bioinform.

[4] Geisler S, Coller J (2013). RNA in unexpected places: long non-coding RNA functions in diverse cellular contexts. Nat Rev Mol Cell Biol.

[5] Chen X, Yan CC, Zhang X, You ZH (2017). Long non-coding RNAs and complex diseases: from experimental results to computational models. Brief Bioinform.

[6] Zhao S, Zhu H, Jiao R, Wu X, Ji G, Zhang X (2020). Prognostic and clinicopathological significance of SNHG6 in human cancers: a meta-analysis. BMC Cancer.

[7] Wang H, Liu Y, Tang A (2020). Prognostic values of long noncoding RNA linc00152 in various carcinomas: an updated systematic review and Meta-analysis. Oncologist.

[8] Ren X, He J, Qi L, Li S, Zhang C, Duan Z, Wang W, Tu C, Li Z (2020). Prognostic and clinicopathologic significance of long non-coding RNA opa-interacting protein 5-antisense RNA 1 in multiple human cancers. Artificial cells, nanomedicine, and biotechnology.

[9] Tani H, Torimura M (2013). Identification of short-lived long non-coding RNAs as surrogate indicators for chemical stress response. Biochem Biophys Res Commun.

[10] Ma Y, Xue Y, Liu X, Qu C, Cai H, Wang P, Li Z, Li Z, Liu Y (2017). SNHG15 affects the growth of glioma microvascular endothelial cells by negatively regulating miR-153. Oncol Rep.

[11] Mi H, Wang X, Wang F, Li L, Zhu M, Wang N, Xiong Y, Gu Y (2020). SNHG15 contributes to Cisplatin resistance in breast Cancer through sponging miR-381. OncoTargets Ther.

[12] Liu K, Hou Y, Liu Y, Zheng J (2017). LncRNA SNHG15 contributes to proliferation, invasion and autophagy in osteosarcoma cells by sponging miR-141. J Biomed Sci.

[13] Ye J, Tan L, Fu Y, Xu H, Wen L, Deng Y, Liu K (2019). LncRNA SNHG15 promotes hepatocellular carcinoma progression by sponging miR-141-3p. J Cell Biochem.

[14] Li Z, Zhang J, Zheng H, Li C, Xiong J, Wang W, Bao H, Jin H, Liang P (2019). Modulating lncRNA SNHG15/CDK6/miR-627 circuit by palbociclib, overcomes temozolomide resistance and reduces M2-polarization of glioma associated microglia in glioblastoma multiforme. J Exp Clin Cancer Res.

[15] Sun X, Bai Y, Yang C, Hu S, Hou Z, Wang G (2019). Long noncoding RNA SNHG15 enhances the development of colorectal carcinoma via functioning as a ceRNA through miR-141/SIRT1/Wnt/beta-catenin axis. Artificial cells, nanomedicine, and biotechnology.

[16] Zhang Y, Zhang D, Lv J, Wang S, Zhang Q (2019). LncRNA SNHG15 acts as an oncogene in prostate cancer by regulating miR-338-3p/FKBP1A axis. Gene.

[17] Li M, Bian Z, Jin G, Zhang J, Yao S, Feng Y, Wang X, Yin Y, Fei B, You Q (2019). LncRNA-SNHG15 enhances cell proliferation in colorectal cancer by inhibiting miR-338-3p. Cancer Med.

[18] Saeinasab M, Bahrami AR, Gonzalez J, Marchese FP, Martinez D, Mowla SJ, Matin MM, Huarte M (2019). SNHG15 is a bifunctional MYC-regulated noncoding locus encoding a lncRNA that promotes cell proliferation, invasion and drug resistance in colorectal cancer by interacting with AIF. J Exp Clin Cancer Res.

[19] Jiang H, Li T, Qu Y, Wang X, Li B, Song J, Sun X, Tang Y, Wan J, Yu Y (2018). Long non-coding RNA SNHG15 interacts with and stabilizes transcription factor slug and promotes colon cancer progression. Cancer Lett.

[20] Liu Y, Li J, Li F, Li M, Shao Y, Wu L (2019). SNHG15 functions as a tumor suppressor in thyroid cancer. J Cell Biochem.

[21] Chen SX, Yin JF, Lin BC, Su HF, Zheng Z, Xie CY, Fei ZH (2016). Upregulated expression of long noncoding RNA SNHG15 promotes cell proliferation and invasion through regulates MMP2/MMP9 in patients with GC. Tumour Biol.

[22] Zhang JHWH, Yang HG (2016). Long noncoding RNA SNHG15, a potential prognostic biomarker for hepatocellular carcinoma. Eur Rev Med Pharmacol Sci.

[23] Cui HXZM, Liu K, Liu J, Zhang ZL, Fu L (2018). LncRNA SNHG15 promotes proliferation and migration of lung Cancer via targeting microRNA-211-3p. Eur Rev Med Pharmacol Sci.

[24] Dong YZMX, Li GS (2018). Long non-coding RNA SNHG15 indicates poor prognosis of non-small cell lung cancer and promotes cell proliferation and invasion. Eur Rev Med Pharmacol Sci.

[25] Jin B, Jin H, Wu HB, Xu JJ, Li B (2018). Long non-coding RNA SNHG15 promotes CDK14 expression via miR-486 to accelerate non-small cell lung cancer cells progression and metastasis. J Cell Physiol.

[26] Du Y, Kong C, Zhu Y, Yu M, Li Z, Bi J, Li Z, Liu X, Zhang Z, Yu X (2018). Knockdown of SNHG15 suppresses renal cell carcinoma proliferation and EMT by regulating the NF-kappaB signaling pathway. Int J Oncol.

[27] Guo XBYH, Wang JY (2018). Evaluating the diagnostic and prognostic value of long non-coding RNA SNHG15 in pancreatic ductal adenocarcinoma. Eur Rev Med Pharmacol Sci.

[28] Kong Q, Qiu M (2018). Long noncoding RNA SNHG15 promotes human breast cancer proliferation, migration and invasion by sponging miR-211-3p. Biochem Biophys Res Commun.

[29] Wu DM, Wang S, Wen X, Han XR, Wang YJ, Shen M, Fan SH, Zhang ZF, Shan Q, Li MQ (2018). LncRNA SNHG15 acts as a ceRNA to regulate YAP1-hippo signaling pathway by sponging miR-200a-3p in papillary thyroid carcinoma. Cell Death Dis.

[30] Huang L, Lin H, Kang L, Huang P, Huang J, Cai J, Xian Z, Zhu P, Huang M, Wang L (2019). Aberrant expression of long noncoding RNA SNHG15 correlates with liver metastasis and poor survival in colorectal cancer. J Cell Physiol.

[31] Qu C, Dai C, Guo Y, Qin R, Liu J (2019). Long noncoding RNA SNHG15 serves as an oncogene and predicts poor prognosis in epithelial ovarian cancer. OncoTargets and therapy.

[32] Adams BD, Parsons C, Walker L, Zhang WC, Slack FJ (2017). Targeting noncoding RNAs in disease. J Clin Invest.

[33] Muller S, Raulefs S, Bruns P, Afonso-Grunz F, Plotner A, Thermann R, Jager C, Schlitter AM, Kong B, Regel I (2015). Next-generation sequencing reveals novel differentially regulated mRNAs, lncRNAs, miRNAs, sdRNAs and a piRNA in pancreatic cancer. Mol Cancer.

[34] Serrati S, De Summa S, Pilato B, Petriella D, Lacalamita R, Tommasi S, Pinto R (2016). Next-generation sequencing: advances and applications in cancer diagnosis. OncoTargets and therapy.

[35] Wang Q, Ding J, Nan G, Lyu Y, Ni G (2019). LncRNA NOC2L-4.1 functions as a tumor oncogene in cervical cancer progression by regulating the miR-630/YAP1 pathway. J Cell Biochem.

[36] Lei H, Gao Y, Xu X (2017). LncRNA TUG1 influences papillary thyroid cancer cell proliferation, migration and EMT formation through targeting miR-145. Acta Biochim Biophys Sin Shanghai.

[37] Wu L, Wang X, Guo Y (2017). Long non-coding RNA MALAT1 is upregulated and involved in cell proliferation, migration and apoptosis in ovarian cancer. Exp Ther Med.

[38] Feng Z, Chen R, Huang N, Luo C (2020). Long non-coding RNA ASMTL-AS1 inhibits tumor growth and glycolysis by regulating the miR-93-3p/miR-660/FOXO1 axis in papillary thyroid carcinoma. Life Sci.

[39] Jafarzadeh M, Soltani BM, Soleimani M, Hosseinkhani S (2020). Epigenetically silenced LINC02381 functions as a tumor suppressor by regulating PI3K-Akt signaling pathway. Biochimie.

[40] Ma X, Mo M, Tan HJJ, Tan C, Zeng X, Zhang G, Huang D, Liang J, Liu S, Qiu X. LINC02499, a novel liver-specific long non-coding RNA with potential diagnostic and prognostic value, inhibits hepatocellular carcinoma cell proliferation, migration, and invasion. Hepatol Res. 2020. 10.1111/hepr.13491 32039538

[41] Ma ZHH, Wang J, Zhou Y, Pu F, Zhao Q, Peng P, Hui B, Ji H, Wang K (2017). Long non-coding RNA SNHG15 inhibits P15 and KLF2 expression to promote pancreatic Cancer proliferation through EZH2-mediated H3K27me3. Oncotarget.

